# Design and Engineering of Deimmunized Vaccinia Viral Vectors

**DOI:** 10.3390/biomedicines8110491

**Published:** 2020-11-11

**Authors:** Kevin Song, Mariya Viskovska

**Affiliations:** Icell Kealex Therapeutics, 2450 Holcombe Blvd Suite J, JALBS@TMC, Houston, TX 77021, USA; Kevinsong0109@gmail.com

**Keywords:** oncolytic vaccinia virus, immunogenicity, deimmunization, neutralizing antibody, complement

## Abstract

Vaccinia viral (VV) vectors are increasingly used in oncolytic virus therapy and vaccine development for cancer and infectious diseases. However, their effectiveness is hindered by the strong anti-viral immune response induced by the viral vector. In this review, we discuss the strategies to deimmunize vaccinia viral vector. One approach is to mask the virus from the neutralization antibody responses by mapping and eliminating of B-cell epitopes on the viral membrane proteins. The recombinant VVs contain one or more viral glycoproteins with mutations in the neutralizing antibody epitopes, resulting in viral escape from neutralization. In addition, a regulator of complement activation (e.g., CD55) can be expressed on the surface of the virus particle, leading to increased resistance to complement-mediated neutralization.

## 1. Introduction

Oncolytic viruses specifically infect, replicate in, and kill tumor cells, while leaving normal cells undamaged. This preference for the transformed cells allows oncolytic viruses to be ideal candidates for the development of new cancer therapies. Various oncolytic viruses have been utilized to employ their tumor-specific killing activities by both direct (e.g., cell lysis due to viral replication and immune-mediated cytotoxicity) and indirect mechanisms (e.g., stimulation of the bystander cell killing, induction of cytotoxicity, etc.) [1,2,3,4,5]. Oncolytic vaccinia virus (VV) is an appealing addition to the current treatment options, demonstrating efficacy and safety in animal models and in early clinical studies [6,7,8,9]. In addition to infecting and killing tumor cells directly, VV may also induce a T-cell response against tumor antigens, increasing the efficiency of the tumor lysis. Whereas in some viruses, this specificity toward cancer cells is naturally occurring (e.g., vesicular stomatitis virus, reovirus, mumps virus), other viruses can be genetically modified to improve their tumor specificity as well as to reduce their ability to induce antiviral immune response (e.g., adenovirus, measles virus, polio, and vaccinia virus) [10,11,12]. In addition, VV can be engineered to express genes that enhance antitumor immunity by recruitment of T cells and macrophages [13,14,15].

The effectiveness of oncolytic viruses is hindered by the strong immune response induced by the virus [16]. With each subsequent administration of the virus, the immune response is faster and stronger, which significantly restricts the ability of the virus to persist long enough to reach the tumor. A direct injection of the virus into the tumor overcomes this limitation and delivers all the viral particles directly to the cancer cells. However, this approach may not be suitable for some tumors and does not take into the account cases in which the tumors may have metastasized to other locations. A more desirable systemic administration of the virus exposes it to the host immune system capable of recognizing and eliminating potential pathogens. Immune factors, such as neutralizing antibodies (NAbs), recognize and bind viral glycoproteins with high affinity and prevent virus interaction with host cell receptors, preventing a successful infection of the cells and marking the virus for destruction either by complement or by other immune cells. Several oncolytic viruses, such as adenovirus, herpes simplex virus, and vesicular stomatitis virus have been genetically attenuated to placate their ability to induce antiviral defenses and improve tumor specificity [12]. Modifications of the antigenic epitopes on the viral surface proteins may significantly limit their recognition by the NAbs. This approach was successful in creating the NAb-escaping variants of the measles virus [17,18], influenza virus [19], hepatitis B and C virus [20], human immunodeficiency virus [21,22], adenovirus, and the adeno-associated vectors (AAVs) [23,24]. In all cases, the combination of the epitope mapping by peptide scanning and the structural analysis of the viral antigens in complex with their binding NAbs aided in mapping out regions and specific residues on these proteins that are important for the NAb binding [25,26]. This review describes the progress in the development of low immunogenic VV vectors.

## 2. Targets for Neutralizing Antibody

Oncolytic vaccinia virus is the most studied member of Poxviridae and is a large, enveloped, dsDNA virus. VV replicates in the cell cytoplasm and encodes more than 200 open reading frames (ORFs) within a 190-kb double-stranded DNA genome. The VV genome can accept as much as 20 kb of foreign DNA, making it ideal as a gene delivery vehicle. Strains highly specific to the tumor cells have been reported [27,28]. The recombinant VV vectors are being developed to deliver eukaryotic genes, such as tumor-associated antigens, to the tumors and thus facilitate an induction of the host immune system directed to kill the cancer cells.

Vaccinia virus infection produces multiple forms of infectious particles, namely intracellular mature virions (IMV), intracellular enveloped virions (IEV), cell-associated enveloped virions (CEV), and extracellular enveloped virions (EEV) [29,30]. IMVs are the most abundant single layer particles inside the cells. These particles remain inside the cells and are released only during cell lysis. Once released, IMVs efficiently infect neighboring cells via interactions between cell receptors and viral glycoproteins imbedded in the IMV membrane. A small number of IMVs is subsequently wrapped with the double layers of Golgi membrane to form IEVs, which are then transported through microtubules to the cell periphery, losing one membrane during virion egress to become CEVs. A smaller number (~5%) of the IMVs avoids the Golgi and moves straight toward the cell’s periphery where it acquires an outer envelope via fusion with the cell plasma membrane and is subsequently released into the extracellular space as EEVs [31,32,33,34]. Thus, EEV is composed of the viral DNA core, the intermediate IMV, and the outermost membrane. This outer membrane can be easily lost, converting EEVs back to the IMVs exposing the IMV imbedded antigens [35].

Many of the poxvirus genomes, including those of different strains of VV, have been sequenced. The genome of the vaccinia virus Western Reserve (WR) strain contains 218 potential ORFs. WR IMVs contain 81 viral proteins, including structural proteins, enzymes, transcription factors, etc. Among glycoproteins imbedded into the viral membrane A27L, H3L, L1R, and D8L have been identified as major immunogenic proteins [25,36,37,38,39,40,41,42]. The IMV proteins A27L, H3L, and D8L serve as the adhesion molecules that bind to host glycosaminoglycans (GAGs) heparan sulfate (HS) and chondroitin sulfate (CS) and mediate endocytosis of the virus into the host cell. The L1R protein is involved in virus maturation.

### 2.1. D8L

VV D8L is an IMV membrane protein expressed early in the infection and is involved in viral adhesion to host cells. While A27L and H3L interact with the HS host cell receptors, D8L binds to the CS receptor via its N-terminal domain (between residues 1–234) [40]. As one of the main viral antigens, D8L elicits a strong NAb response. Several NAbs targeting the D8L protein have been described. These Abs are separated into four main specificity groups. ELISA mapping revealed that group II antibodies target a linear epitope (residue 91–110) [37]. Alanine scanning and point mutation analysis (PMA) further narrowed down the epitopes to 10 specific residues within the peptide: H95, W96, N97, K99, Y101, S102, S103, E106, H110, and D112 [37]. Mutations of these residues to alanine resulted in a significant reduction in Ab binding to D8L. Groups I, III, and IV antibodies target conformational epitopes and the binding residues were identified by electron microscopy. Group I antibodies target residues N9, I10, E11, T12, K13, K14, D75, Y76, H80, L81, I82, D83, V84, Y85, K86, Y87, S88, G89, E90, Q122, L124, D125, K163, T187, P188, and N190. Group III antibodies target residues E30, T34, T35, R44, N46, F47, K48, G49, G50, Y51, N59, E60, V62, L63, S64, H95, W96, N97, Y101, S103, Y104, E105, E106, and K108. Group IV antibodies target residues Q3, L5, T39, G40, K41, R44, K108, N145, I174, N175, H176, S177, S204, L205, I215, E217, Y219, R220, N221, Y223, K224, and N226 [37]. Importantly, D8L knockout VVs in the strains WR and LIVP have been successfully generated. The study showed that removing D8L from the viral particles has no effect on the pathogenicity of the virus in an intracutaneous infection of rabbits, and it fails to infect the rat brain, indicating the D8L function is connected to neural tissues and its interaction with CSs might not be the main infection route [43].

### 2.2. L1R

L1R is a transmembrane protein found on the surface of the mature IMV particles. Its transmembrane domain lies in the C-terminal of the protein between residues 186 and 204 [44]. L1R is encoded by the L1R ORF, is highly conserved, and plays an essential role in viral entry and maturation [44,45]. Several studies were successful in identifying the NAb binding epitopes on the L1R. A study by Su et al. identified potent NAbs recognizing a linear epitope spanning residues 118–128 and a conformation epitope that partially overlapped with the linear peptide and comprised of residues K125 and K127 [25]. A more recent study identified a group of three anti-L1R monoclonal Abs that potently neutralized VV in an isotype- and complement-independent manner. All three NAbs recognized a conformational epitope with D35 as the key residue [39]. Viral clones that contained a single amino acid mutation at residue D35 (either D35N or D35Y substitution) were completely resistant to neutralization by all NAbs, indicating that D35 is essential for NAb recognition of L1R. The D35N escape mutant was also described earlier by Ichihashi et al. [44]. In addition to D35, Kaever et al. identified residues E25, N27, Q31, T32, K33, S58, D60, and D62 directly involved in binding with the NAb [39].

### 2.3. A27L

A27L functions in viral host cell recognition and entry, albeit via a different receptor. A27L binds to the HSs expressed on the host cells via its N-terminal domain (residues 21 to 30) and is attached to the IMV envelope by interacting with the envelope protein A17 through its C-terminal domain. A recent study has identified several linear epitopes on the A27L that are recognized by the anti-A27L NAbs [38]. The Abs were categorized into four different groups with the Abs in group I binding to the peptide (residues 31 to 40) adjacent to the HS binding site and showing potent virus neutralization in the presence of complement. Crystal structures of the full-length A27L in a complex with these Abs identified residues E33, I35, V36, K37, and D39 to be critical for binding. Alanine substitutions for these residues resulted in the decreased ability of the Abs to bind to the peptide. A further analysis of the structures showed that residues K27, A30, R32, A34, E40, R107, P108, and Y109, although not critical, also contributed to the A27L–NAb binding [38]. VV with A27L deletion or inactivation is viable, while the virus forms smaller plaque [46].

### 2.4. H3L

**Figure 1:**
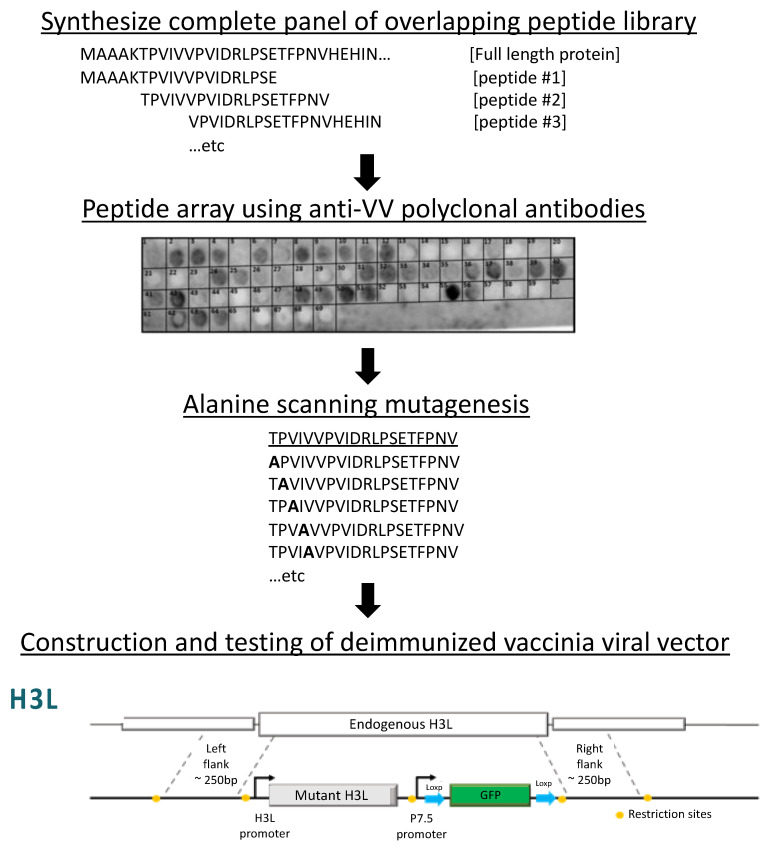
Schematic diagrams for an experimentally driven B cell epitope deletion strategy. A panel of overlapping synthetic peptide fragments spanning the full sequence of H3L viral protein is synthesized. The peptides are then tested for immune recognition, typically using in vitro ELISA with polyclonal anti-vaccinia viral (VV) antibodies. Identified immunogenic peptides are subjected to alanine scanning mutagenesis and retested for immune recognition. Confirmed deimmunizing mutations are then engineered back into the H3L viral proteins and tested for viral packaging, production, stability, and activity. This figure describes the B cell epitope deletion procedure that is conducted in the author’s group [52].

H3L is the membrane protein tethered to the membrane of the mature IMV particles post-translationally via its hydrophobic region in the C-terminus [47,48]. It is expressed late during the infection and, like the A27L, recognizes the HS cell surface receptors and plays a major role in VV adhesion to the cells [48]. H3L is an immunodominant antigen in the anti-VV Ab response and a direct target of NAbs in humans immunized by the smallpox vaccine [41,42,49]. Strong immune responses to H3L have also been shown in mice and rabbits [50,51]. Our studies have shown that peptide array (Figure 1) identified several linear epitopes on the H3L that are recognized by the anti-VV polyclonal neutralizing antibodies [52]. Further analysis by alanine scanning identified total of 21 residues positive for Ab binding: I14, D15, R16, K38, N40, E45, V52, E131, T134, F135, L136, R137, R154, E155, I156, I198, E250, K253, P254, N255, and F256 [52].

## 3. Mutant Virus Library Screening

One approach to identify low immunogenic VVs is to isolate neutralization escape mutants from a mutant virus library. For example, to map the epitopes of the anti-L1R neutralizing antibodies, a mutant VV (WR strain) library was generated from HeLa cells that were infected with WR VV in the presence of a potent chemical mutagen ethyl methanesulfonate (EMS) [39]. The resulting mutant viral stock was incubated with anti-L1R MAb at a final concentration of 100 µg/mL for 1 h before reinfection of HeLa cells. This selection was repeated 3 to 4 times with a constant antibody concentration but with increasingly less virus concentration than the previous round. The final virus clones were resistant to anti-L1R Ab neutralization in vitro. Sequencing the L1R gene expressed by these escape variants identified the D35 as the key epitope of the NAbs [39].

The same approach was used by our group to identify NAb epitopes on H3L, A27L, and D8L proteins [52]. A stock of mutant VVs was prepared from CV-1 cells that were infected with the WR VV in the presence of EMS to induce transition mutations in viral DNA. Polyclonal anti-VV antibodies were used to screen the mutated virus pool for escape mutants. The mutant viral stock was incubated with the polyclonal anti-VV antibodies for 1 h and then used to infect the CV-1 cells. During the first round of infection, the titer of the mutant virus was significantly reduced by the Abs. After multiple rounds of infections with constant Ab concentration and with the increasingly more purified virus than the previous rounds, the passaged viral stock was no longer significantly neutralized by the Abs. A clone of the escape mutant was plaque-purified and showed a significant escape of neutralization by anti-VV antibodies. Viral DNA from pure virus was isolated and PCR was used to amplify the A27L, H3L, and D8L genes, the major Ab antigens of the VV. PCR products were sequenced and showed presence of the mutations in genes A27L, D8L, and H3L. The D8L coding sequence had a nucleotide insertion at residue I278. The A27L coding sequence showed two mutations at residues I35 and D39 that were also identified previously as the NAb epitopes [38]. The H3L sequence showed a single amino acid substitution at residue P44, indicating that it may be critical in the H3L interactions with the Abs [52].

## 4. Antibody Epitope Deletion

Once the escape mutations were identified, a recombinant VV was generated in our group by homologous recombination to introduce the mutations into the viral genes encoding proteins (Figure 1) [52]. For each recombinant protein, a DNA fragment containing the viral gene’s native promoter, ORF (with appropriate mutations), and approximately 250-bp flanking regions on either side to promote homologous recombination into the appropriate gene in the VV genome was synthesized and cloned into a shuttle plasmid. A green fluorescent protein (GFP) expression cassette under the control of the VV p7.5 promoter and flanked by LoxP sites was inserted immediately downstream of the gene stop codon before the right flanking sequence. The plasmids were transfected into the CV-1 cells and allowed to recombine with the VV genome. The fluorescence marker expressed from the GFP cassette was used to purify clones and after purification GFP was removed using the LoxP sites. The correct gene insertion into the VV genome was verified by PCR and gene sequencing. With the addition of each plasmid rounds of screening, purifications were performed, followed by PCR and sequencing to make sure that the correct mutations were present. GFP was removed before the recombination with each subsequent plasmid [52].

After four rounds of recombination, the final neutralization escape variant containing modifications in all four proteins was tested in vitro for the resistance to anti-VV neutralization by the plaque reduction neutralization test (PRNT) using a panel of five polyclonal anti-VV NAbs. NAbs were preincubated with either the escape variant or the wildtype VV (control) in the presence of sterile baby rabbit complement before infecting the CV-1 cells and determining the titers in each treatment group by plaque assay. The neutralization escape variant showed a significantly lower neutralization by the Abs when compared to the control virus. Additionally, a virus replication assay showed that the recombinant virus was significantly more efficient in replicating in the presence of neutralizing Ab, compared to the control [52].

## 5. Overcoming Complement-Mediated Virus Neutralization

**Figure 2:**
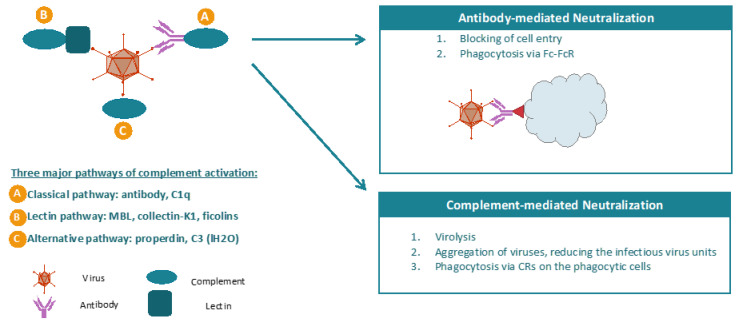
The mechanisms of antibody- and complement-mediated neutralization of virus. Antibody can block cell entry of virus. In addition, antibody can lead to phagocytosis of the virus via Fc–FcR interaction. The complement system is activated primarily by three pathways, including classical pathway, lectin pathway, and alternative pathway. In the classical pathway, viral antigen is recognized by neutralization antibody. The viral antigen–antibody complex induces activation of complement system. Neutralization of viruses by complement occurs owing to different mechanisms. First, virus can form a membrane attach complex (MAC) on the viral envelopes and produce holes of ~100 A diameter in the virus membrane. In addition, opsonization of viral surface with complement can lead to aggregation of viruses as well as phagocytosis of these viruses via a complement receptor (CR) present on the phagocytic cells.

Complement is a key component of the innate immune system, targeting the virus for neutralization and clearance from the circulatory system [53,54,55,56]. The complement system can be activated by three major pathways, including classical pathway (CP), lectin pathway (LP), and alternative pathway (AP). Vaccinia virus mainly activates complement through CP pathway by forming antigen–antibody complexes that are recognized by the C1 complex. Complement could enhance neutralization antibody’s neutralizing efficacy, and antibody-mediated protective immunity induced by smallpox vaccination was largely decreased in vitro in the absence of complement, indicating the critical role of complement in the neutralization of VV (Figure 2). Complement activation results in cleavage and activation of C3 and deposition of opsonic C3 fragments on surfaces. Subsequent cleavage of C5 leads to assembly of the membrane attack complex (C5b, 6, 7, 8, 9), which disrupts lipid bilayers.

Complement activation can be negatively regulated by several membrane regulators of complement activation (RCAs). RCAs downregulate complement activation at different steps. First, CD35 (complement receptor 1) and CD55 (decay-accelerating factor) inhibit the formation and accelerate the decay of C3 convertases (C3-activating enzymes). Second, CD35 and CD46 (membrane cofactor protein) catabolizes C4b and C3b, inhibiting formation of the C3 convertases C4b2a and C3bBb. Third, CD59 prevents the formation of the membrane attack complex [57,58].

Studies have shown that the extracellular enveloped form of VV (EEV) is resistant to complement because of incorporation of host RCA into its envelope [59]. Cellular membrane proteins such as CD46, CD55, CD59, CD71, CD81, and MCH I have been detected in EEV but not IMV. In addition, EEV produced from rat cells expressing the human RCA were more resistant to human complement when compared to EEV from control rat cells that express neither CD55 nor CD59. In addition, the expression of CD55 alone endows the VV with ability of resisting complement significantly, indicating that CD55 is the major RCA for VV [59].

Our studies have shown that recombinant VV virions encoding CD55 has the ability to modulate complement activation and reduce complement-mediated virus neutralization as compared to the wild-type virus [52]. CD55 is expressed on the viral membrane as a fusion protein with A27L protein under the transcriptional control of A27L promoter. First, the ability of CD55-VV to escape complement-mediated neutralization was investigated. This was done by adding CD55-VV or VV control to the CV-1 cells in the presence of 1:10 human complement and using heat-activated complement as control to calculate the escape rate. The results suggested that CD55-VV is able to escape complement-mediated neutralization more effectively than NEV. The ability of CD55-expressed mutant VV variant to escape the neutralization of complement with anti-VV polyclonal Abs was further investigated. The results suggested that CD55-expressed mutant VV variant escaped the neutralization more effectively than control virus in the presence of complement and neutralization antibodies. Based on these results, it is concluded that the CD55 expressing VV can efficiently escape complement/Nab-mediated neutralization in vitro [52].

## 6. Conclusion Remarks

Oncolytic VV has shown promising results in clinical studies, where it was discovered that immunogenicity was a major obstacle in effectively treating patients with normal immune systems. To overcome this limitation, deimmunization strategies can be employed to develop a low immunogenic vaccinia viral vector by incorporating one or more mutations in the genes encoding proteins involved in binding neutralization antibodies or T cells or by expressing a complement activation modulator. These modifications result in novel VVs having the ability to escape VV-specific neutralization antibodies or T cells when compared to the wild-type virus.
